# Authors' Reply

**DOI:** 10.1371/journal.pmed.0030210

**Published:** 2006-04-25

**Authors:** Frits Rosendaal, Pieter H Reitsma

**Affiliations:** **1**Leiden University Medical CenterLeidenNetherlands; **2**Academic Medical CenterAmsterdamNetherlands

William Steinsmith is correct that the odds ratio (OR) is used to approximate the relative risk, but incorrect that in this instance, and most other ones, the two would differ by more than a trivial amount, since he has failed to appreciate the case-control design.

Indeed, if the cumulative risk of disease is p1 in the exposed and p0 in the nonexposed, the relative risk (p1/ p0) only equates the OR, [p1/(1-p1)]/[p0/(1-p0)], when exposure confers no excess risk. However, in a case-control study, p1 and p0 cannot be directly estimated (nor, obviously, can their odds), and the OR is the only possible estimator (hence its frequent use).

We will illustrate the theory starting with a cohort study, and then moving from this to a case-control study. When risks are low, the OR will always be a good approximation of the relative risk because OR = (p1/p0)/[(1-p1)/(1-p0)], in which the second part of the term will be close to one. So, suppose a trait is present in 20% of a population, with a risk of disease of 1% in those without the trait, and 2.5% in those with the trait, i.e., a relative risk of 2.5. These are likely to be close to the actual annual numbers for risk of haemorrhage under anticoagulant treatment with and without the *VKORC1* variant [ 1, 2]. If we follow 10,000 people for one year—8,000 without the trait, of whom 80 will develop disease; 2,000 with the trait, of whom 50 will develop disease—RR = (50/2,000)/(80/8,000) = 2.5000, and OR = (50/1,950)/(80/7,920) = 2.5385.When the OR is written out in full to [(50/2,000)/(1,950/2,000)]/[(80/8,000)/(7920/8 000)], this can easily be reduced to the above.

In a case-control study, all cases are included, but there are only a fraction of all noncases (controls). With a sampling fraction of 1/10, the case-control study sampled from this cohort would look like the following: 80 cases without and 50 cases with the trait, 792 controls without and 195 controls with the trait (OR = [50/195]/[80/792] = 2.5385).

With a sampling fraction of 1/100, there would be 79.2 unexposed and 19.5 exposed controls, and the OR would still be 2.54. This demonstrates that the actual risk or odds of disease cannot be derived once only a sample of individuals without disease are included, but that the ratio of exposed over unexposed controls (195/792) remains valid whatever the sampling fraction. This has been called the “exposure odds”, and many prefer to write the OR as the exposure odds ratio: OR = (50/80)/(195/792) = 2.54.

In a cohort study, the OR can be easily recalculated into a risk ratio (RR), since the actual risks (p0 and p1) are known [ 3]: RR = OR/[(1-p0) + p0*OR] [ 3]. In the example above, RR = 2.5385/(0.99 + 0.01*2.5385) = 2.5000.

In a case-control study, because the number of controls is only a fraction of the actual number of individuals without disease in the cohort, absolute risks cannot be calculated, and a recalculation from OR to RR is not possible (unless there is external information on the absolute risks).

This implies that it is not possible to calculate from our data how different the OR was from the RR, as Steinsmith tried. We can, however, in this particular case, make an estimate, since we know the risk of haemorrhage under anticoagulant treatment from previous studies to be around 1% per year. With a background risk of 1% per year, all the ORs mentioned in our paper are within 2% of the relative risk. The highest OR of 2.6 (2.5641) would relate to a relative risk of 2.5 (2.5246)—a trivial difference. Steinsmith's further suggestions for analyses, i.e., to use likelihood ratios, are relevant to studies of diagnostic tests in which the aim is to evaluate the presence or absence of disease. This is not the analysis one would use in aetiologic studies such as ours.

Generally, since most diseases are infrequent, ORs are good estimators of relative risks under this “rare disease assumption”. For a disease with a frequency of 10%, which is high, the difference between OR and RR is still only 10%. On a higher theoretical level, one could argue that the parameter to estimate is not the relative risk, but the rate ratio, i.e., the ratio of two incidence rates. While a cumulative risk is a probability, an incidence has time -1 as its unit, and lies between zero and infinity. Since the incidence rate is the basic measure of disease occurrence, the rate ratio is the prime comparator, to be preferred over relative risks (which, over time, will converge to unity, because, to quote John Maynard Keynes, “in the long run we are all dead”). It can be shown that under certain sampling conditions, i.e., when controls are sampled from a dynamic population, there is no need for the “rare disease assumption”, and the OR is the exact equivalent of an incidence rate ratio [ 4].
